# Derivation and validation of urinary TIMP-1 for the prediction of acute kidney injury and mortality in critically ill children

**DOI:** 10.1186/s12967-022-03302-0

**Published:** 2022-02-23

**Authors:** Hui Huang, Qiang Lin, Xiaomei Dai, Jiao Chen, Zhenjiang Bai, Xiaozhong Li, Fang Fang, Yanhong Li

**Affiliations:** 1 Department of Nephrology and Immunology, Children’s Hospital of Soochow University, Suzhou, JiangSu Province China; 2 Pediatric Intensive Care Unit, Children’s Hospital of Soochow University, Suzhou, JiangSu Province China; 3 Institute of Pediatric Research, Children’s Hospital of Soochow University, Suzhou, JiangSu Province China; 4 Department of Nephrology and Immunology, Institute of Pediatric Research, Children’s Hospital of Soochow University, Suzhou, JiangSu Province China

**Keywords:** Acute kidney injury, Critically ill children, Mortality, Urinary TIMP-1

## Abstract

**Background:**

Acute kidney injury (AKI) is associated with high morbidity and mortality. Multiple urinary biomarkers have been identified to be associated with the prediction of AKI and outcomes. However, the accuracy of these urinary biomarkers for AKI and associated outcomes has not been clearly defined, especially in heterogeneous populations. The aims of the study were to compare the ability of 10 existing or potential urinary biomarkers to predict AKI and pediatric intensive care unit (PICU) mortality and validate urinary tissue inhibitor of metalloproteinases-1 (uTIMP-1) as a better biomarker for early prediction in heterogeneous critically ill children.

**Methods:**

A derivation-validation approach with separate critically ill cohorts was designed. We first conducted a prospective cohort study to determine the ability of 10 urinary biomarkers serially measured in 123 children during the first 7 days of PICU stay to predict AKI and PICU mortality (derivation study) and further validated the better biomarker of uTIMP-1 in a separate cohort of 357 critically ill children (validation study). AKI diagnosis was based on KDIGO classification with serum creatinine and urine output. PICU mortality was defined as all-cause mortality.

**Results:**

In the derivation cohort, 17 of 123 (13.8%) children developed AKI stage 3 or died during the PICU stay, and both the initial and peak uTIMP-1 displayed the highest AUCs of 0.87 (0.79–0.94) and 0.90 (0.84–0.96), respectively, for predicting AKI stage 3 or death. In the validation cohort, 78 of 357 (21.8%) developed AKI during the first week after admission, and 38 (10.6%) died during the PICU stay. The initial uTIMP-1 level was validated to be independently associated with AKI (AOR = 2.88, 95% CI 1.97–4.21), severe AKI (AOR = 2.62, 95% CI 1.78–3.88), AKI stage 3 (AOR = 2.94, 95% CI 1.84–4.68) and PICU mortality (AOR = 1.92, 95% CI 1.11–3.30) after adjustment for potential confounders. The predictive values of uTIMP-1 for AKI, severe AKI, AKI stage 3 and PICU mortality were 0.80 (0.74–0.86), 0.83 (0.77–0.89), 0.84 (0.77–0.92) and 0.83 (0.76–0.89), respectively.

**Conclusions:**

Urinary TIMP-1 levels have been identified and validated to be independently associated with AKI and PICU mortality in independent prospective cohorts and may be an early potential indicator of AKI and PICU mortality in critically ill children.

**Supplementary Information:**

The online version contains supplementary material available at 10.1186/s12967-022-03302-0.

## Introduction

Acute kidney injury (AKI) is a common clinical complication and is associated with high morbidity and mortality in critically ill patients [1, 2]. Thus, early and accurate diagnosis of AKI is crucial to initiate timing therapeutic intervention to potentially improve clinical outcomes [3]. During recent decades, multiple urinary biomarkers, characterized as noninvasive and early indicators of AKI, have been identified, and various attempts have been made to associate the concentrations of urinary biomarkers with the prediction of AKI and outcomes in various clinical settings [4–6]. However, the accuracy of these urinary biomarkers in the clinical diagnosis of AKI and in the perdition of associated patient outcomes, especially in more heterogeneous populations such as general intensive care unit (ICU) patients, has not been clearly defined [3, 7–9]. None of these urinary biomarkers is routinely used in pediatric clinical practice and adds a clear value beyond the traditional approach in clinical decision making in children, especially in critically ill children, with AKI.

The possible reasons for suboptimal biomarker performance in the critical care setting might be that AKI is a heterogeneous clinical syndrome and has multiple etiologies and variable pathogenesis [3, 10]. The population of pediatric ICUs (PICUs) is also heterogeneous, and AKI etiology and timing are largely unknown. Here, we designed a derivation-validation approach with separate critically ill cohorts and report the results of the prospective investigation in which 10 existing or potential urinary biomarkers, including neutrophil gelatinase-associated lipocalin (NGAL), kidney injury molecule-1 (KIM-1), tissue inhibitor of metalloproteinase-2 (TIMP-2), insulin-like growth factor-binding protein 7 (IGFBP7), [TIMP-2]•[IGFBP7], fatty-acid-binding protein-1 (FABP-1), tissue inhibitor of metalloproteinase-1 (TIMP-1), renin, interferon inducible protein-10 (IP-10) and trefoil factor-3 (TFF-3), were compared in critically ill children for predation of AKI and PICU mortality; and urinary TIMP-1 (uTIMP-1), as the better biomarker, was identified and subsequently validated in an independent cohort of heterogeneous critically ill children for early prediction of AKI and adverse outcome.

## Methods

### Study design and population

We conducted a two-stage prospective cohort study in which we first collected urine samples from a cohort to identify the best biomarker for the prediction of AKI and PICU mortality among 10 candidate urinary biomarkers, including novel potential candidates and previously described biomarkers (derivation study). A separate independent cohort was used to validate the performance value of the best biomarker identified from the derivation (validation study).

**Fig. 1:**
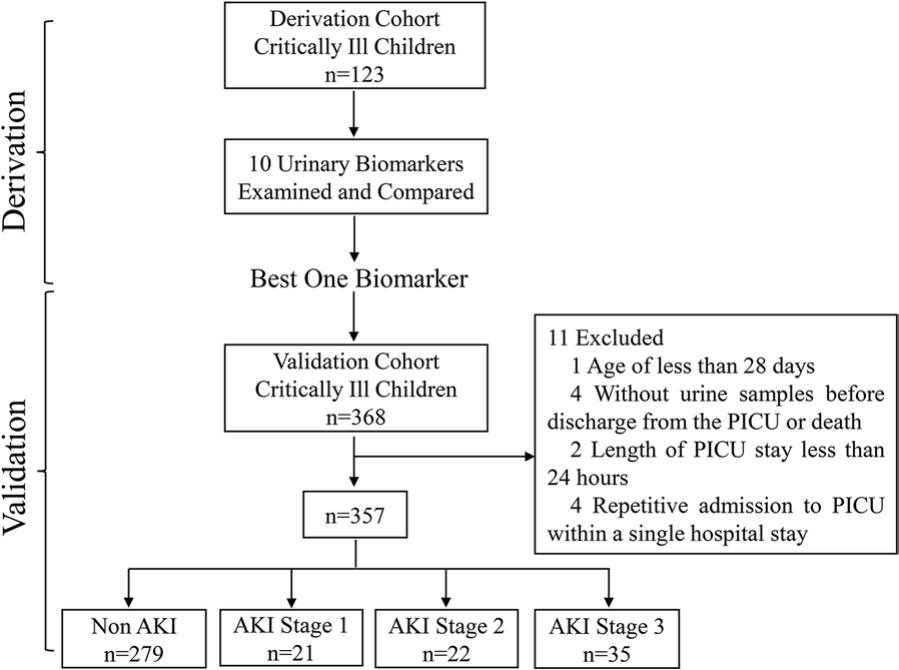
A flow chart representing study design. *AKI* acute kidney injury, *PICU* pediatric intensive care unit

The overall study design is shown in Fig. 1. Both cohorts were conducted in the PICU of a single tertiary children’s hospital and included critically ill children aged between 1 month and 18 years. The derivation cohort was conducted from September to December 2016, and the validation cohort was performed from December 2017 to January 2018 and September to December 2019. The exclusion criteria were as follows: known congenital abnormality of the kidney and a failure to collect urine samples before discharge from the PICU or death. Children had multiple PICU admissions within a single hospital stay, and only their last admission was included in the analysis. The study was approved by the Institutional Review Board at the Children’s Hospital of Soochow University and performed in accordance with the Declaration of Helsinki. Written consent forms were obtained from their parents involved in this study.

### Clinical data collection

In both derivation and validation cohort studies, the medical records of eligible patients were reviewed. Demographic characteristics, including age, body weight and sex, admission diagnosis, clinical status as defined by illness severity, medication and therapeutic interventions, were recorded daily until PICU discharge or death. Sepsis, multiple organ dysfunction syndrome (MODS), shock and disseminated intravascular coagulation (DIC) that developed during the PICU stay were diagnosed by the treating physicians, according to the criteria described previously [11].

### Assessment of illness severity

The score of the pediatric risk of mortality III (PRISM III), which was calculated on the day of PICU admission, was used to assess illness severity of critically ill children in both derivation and validation cohorts, according to methods described in the original study [12] and in accordance with our previous studies [11, 13].

### Diagnosis of AKI

The diagnosis of AKI was based on an increase in serum creatinine (sCr) and/or a reduction in urine output within the first 7 days after PICU admission, according to the criteria of Kidney Disease: Improving Global Outcome (KDIGO) [14]. Baseline sCr was defined as the lowest level obtained within 3 months prior to PICU admission [2]. If the baseline sCr was unknown, the child was assumed to have an estimated glomerular filtration rate (eGFR) of 120 ml/min/1.73 m^2^ [15–17], and baseline sCr was back calculated using the modified Schwartz formula, where eGFR = (0.413 × height)/sCr [18, 19]. The sCr level after PICU admission was measured daily during the first week, followed by routine measurement every 48–72 h during the PICU stay. The severity of AKI was characterized by KDIGO staging, and KDIGO stages 2 and 3 were defined as severe AKI.

### Clinical outcomes

PICU mortality, as the clinical outcome, was defined as all-cause mortality occurring during the PICU stay, including death resulting from withdrawal of therapy.

### Urine sample collection

In the derivation cohort, urine samples were collected within the first 24 h after PICU admission, followed by every 48–72 h during the first 7 days of the PICU stay. In the validation cohort, urine samples were only collected within 24 h after PICU admission. All acquired urine samples were collected using a plastic bag and immediately frozen and stored at − 80 °C. The samples were centrifuged at 1500 *g* at 4 °C for 10 min, and the supernatants were aliquoted for the measurement.

### Measurement of urinary biomarkers

In the derivation cohort study, six biomarkers (KIM-1, FABP-1, TIMP-1, renin, IP-10 and TFF-3) in urine were measured using multiplex bead assays incorporated in human kidney injury panel 1 (HKI1MAG-99K, MILLIPLEX MAP kit, Millipore, Billerica, USA) run on the Luminex FlexMAP 3D instrument according to the manufacturer’s instructions. The calibration curve was calculated using a five-parameter logistic fit, and the concentration of urinary biomarkers was determined. Human Lipocalin 2/NGAL (ab113326, Abcam, USA), TIMP-2 (DY971, R&D Systems, USA) and IGFBP7 (DY1334-05, R&D Systems, USA) ELISA kits were used for the measurement of NGAL, TIMP-2 and IGFBP7 in urine. In the ELISAs, the samples were diluted tenfold to 1000-fold in Reagent Diluent to ensure that the enzymatic reaction was maintained within the linear range. The intra-assay and inter-assay coefficients of variation within and between ELISA tests were < 10%. In the validation cohort, the concentration of uTIMP-1 was measured by ELISA (DTM100, R&D Systems, USA). The minimum detectable level of TIMP-1 was < 0.08 ng/mL, and the coefficient of variation of intra-assay and inter-assay were < 5% and 4.9%, respectively.

In both derivation and validation cohort studies, the concentration of urinary biomarkers was expressed in nanograms per milligram of urinary Cr (ng/mg uCr). The uCr level from the aliquoted sample was measured automatically on an automatic biochemical analyser (Hitachi 7600, Tokyo, Japan) by using the sarcosine oxidase method. For urinary [TIMP-2]•[IGFBP7], the concentrations of TIMP-2 and IGFBP-7 in the urine were multiplied and then divided by 1000 to convert them into international general units, (ng/mL)^2^/1000, in accordance with our previous study [20] and study by others [21].

In addition, the initial and peak values of urinary biomarkers were used for data analysis in the derivation study. For each child, the level of urinary biomarkers from the sample collected in the first 24 h after PICU admission was denoted as the initial value. The highest level among collected samples during the first 7 days after PICU admission was denoted the peak value.

### Statistical analysis

SPSS statistics software Version 22 and GraphPad software Inc. Prism Version 8 was used for statistical analyses. Continuous data are presented as the median and interquartile range (IQR), as they were not normally distributed. Categorical data are presented as counts and percentages. Continuous variables among groups were compared using the Mann–Whitney U test or Kruskal–Wallis H test, and categorical variables were compared using the Chi-square test or Fisher’s exact test, as appropriate. Univariate and stepwise multivariate linear regression analyses were performed to investigate factors potentially associated with the levels of uTIMP-1 in the validation cohort. Multicollinearity of variables was evaluated via tolerance and variance inflation factor (VIF), and tolerance ≤ 0.5 and VIF value ≥ 2 indicated the presence of multicollinearity. In both the derivation and validation cohorts, multivariate logistic regression analyses were performed to investigate the associations between urinary biomarkers and AKI and PICU mortality after adjustment for potential confounders. The Hosmer–Lemeshow goodness-of-fit test was used to evaluate the model fit. Subsequently, the predictive values of urinary biomarkers for AKI and PICU mortality were assessed by receiver operating characteristic (ROC) curves. The area under the ROC curve (AUC) with the corresponding 95% confidence interval (CI) was recorded. In the validation cohort, the predictive accuracy was further assessed by sensitivity, specificity, positive predictive value (PPV) and negative predictive value (NPV) at the optimal cut-off values, which were determined by the maximum Youden index. For all analyses, a two-tailed P < 0.05 was considered significant.

## Results

### Derivation cohort characteristics

The derivation cohort study involved 123 critically ill children. Of a total of 125 children admitted to the PICU during the study period, 2 children had multiple PICU admissions within a single hospital stay, and only their last admission was included in the analysis. The leading cause of PICU admission in the cohort was respiratory diseases (30.9%), followed by neurologic diseases (14.6%), poison/trauma/accident (14.6%) and hematologic diseases (11.4%). None of the children had any known congenital abnormality of the kidney and received aminoglycosides during the PICU stay.

Of the 123 children, 29 (23.5%) developed AKI during the PICU stay, including 16 with AKI stage 1, 8 with stage 2, and 5 with AKI stage 3. All the AKI occurred during the first week after PICU admission. The comparison of the demographic and clinical characteristics and the initial and peak levels of urinary biomarkers among children with non-AKI and AKI stage 1, 2, and 3 is displayed in Additional file 1: Table S1. The PICU mortality in the whole cohort with or without AKI was 15 (12.2%). The comparison of the characteristics and the levels of urinary biomarkers between survivors and non-survivors is displayed in Additional file 1: Table S2.

### Association of urinary biomarkers with AKI stage 3 or death in the derivation cohort

Since there was no significant difference in the initial levels of urinary biomarkers among children with non-AKI, AKI stage 1 and AKI stage 2 (except KIM-1 and TIMP-2), as shown in Additional file 1: Table S1, the comparison of the demographic and clinical characteristics and the initial and peak levels of urinary biomarkers among survivors with non-AKI (n = 86), survivors with AKI stage 1 or 2 (n = 20), and survivors with AKI stage 3 or non-survivors (n = 17) is displayed in Additional file 1: Table S3. The distributions of the initial and peak levels of urinary biomarkers among these groups are displayed in Additional file 2: Fig. S1a, b. In addition, the association of urinary biomarkers with AKI stage 3 or death developed during the PICU stay was analysed in Additional file 1: Table S4 by using univariate and multivariate logistic regression analyses.

### Comparison of urinary biomarkers in predicting AKI stage 3 or death in the derivation cohort

**Fig. 2:**
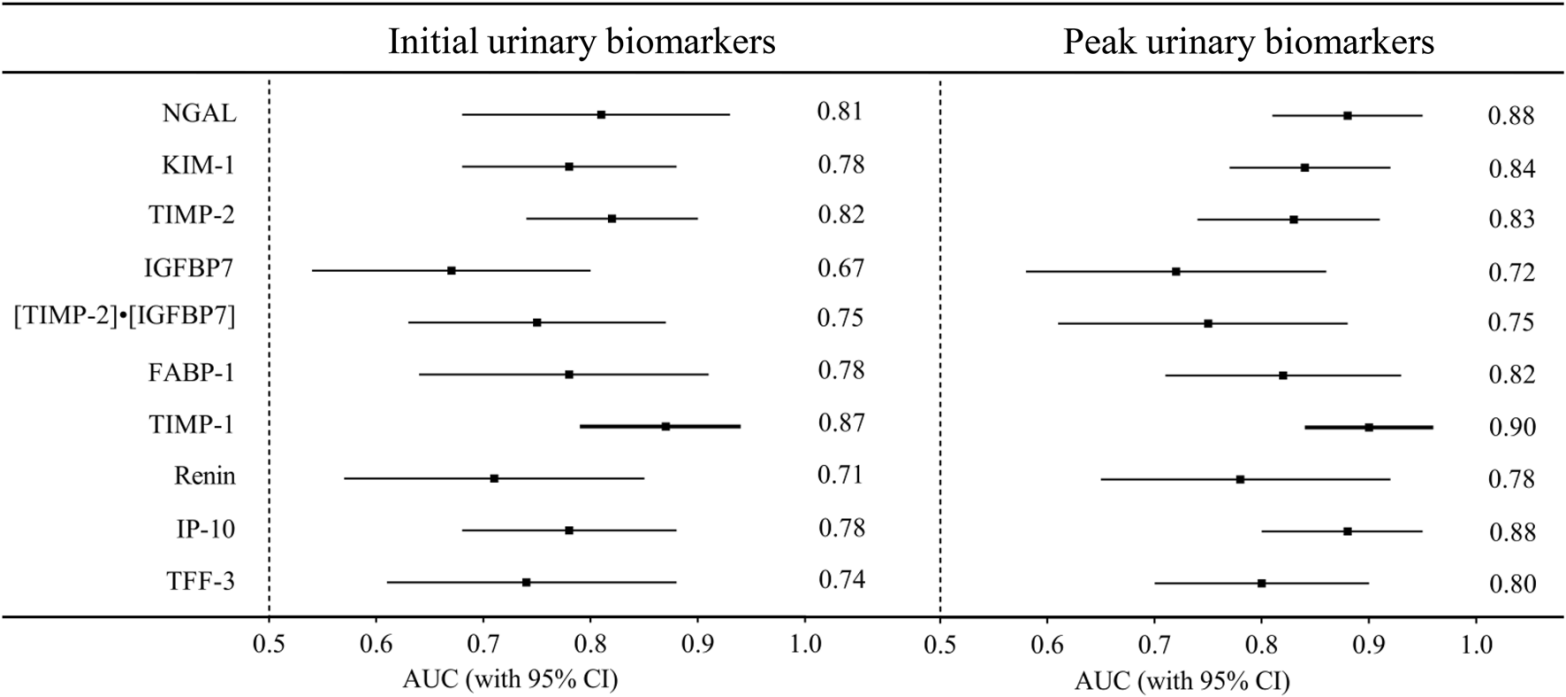
Predictive characteristics of urinary biomarkers for AKI stage 3 or death in the derivation cohort. Boxes and lines represent AUCs and associated 95% confidence intervals, respectively. *AKI* acute kidney injury, *AUC* the area under the ROC curve, *CI* confidence interval, *FABP-1* fatty acid binding protein 1, *IGFBP7* insulin-like growth factor-binding protein 7, *IP-10* interferon inducible protein-10, *KIM-1* kidney injury molecule-1, *NGAL* neutrophil gelatinase-associated lipocalin, *TFF-3* trefoil factor-3, *TIMP-1* tissue inhibitor of metalloproteinases-1, *TIMP-2* tissue inhibitor of metalloproteinases-2

The performance of both initial and peak urinary biomarkers in predicting AKI stage 3 or death developed during the PICU stay is displayed in Fig. 2 and Additional file 1: Table S5. As shown in Fig. 2, both the initial and peak uTIMP-1 displayed the highest AUCs of 0.87 (95% CI 0.79–0.94, P < 0.001) and 0.90 (95% CI 0.84–0.96, P < 0.001) for the prediction. Therefore, the associations between uTIMP-1 levels and AKI or PICU mortality were sought to confirm in the validation study.

### Validation cohort characteristics

The validation cohort study involved 357 critically ill children. Of a total of 368 children admitted to the PICU during the study period, 11 were excluded because of age of less than 28 days, a failure to collect urine samples before discharge from the PICU or death and repetitive admission to the PICU within a single hospital stay, as displayed in Fig. 1. The leading cause of PICU admission in the validation cohort was respiratory diseases (42.4%), followed by neurological diseases (16.1%), hematologic/oncologic diseases (9.9%) and gastrointestinal diseases (9.7%).

**Fig. 3:**
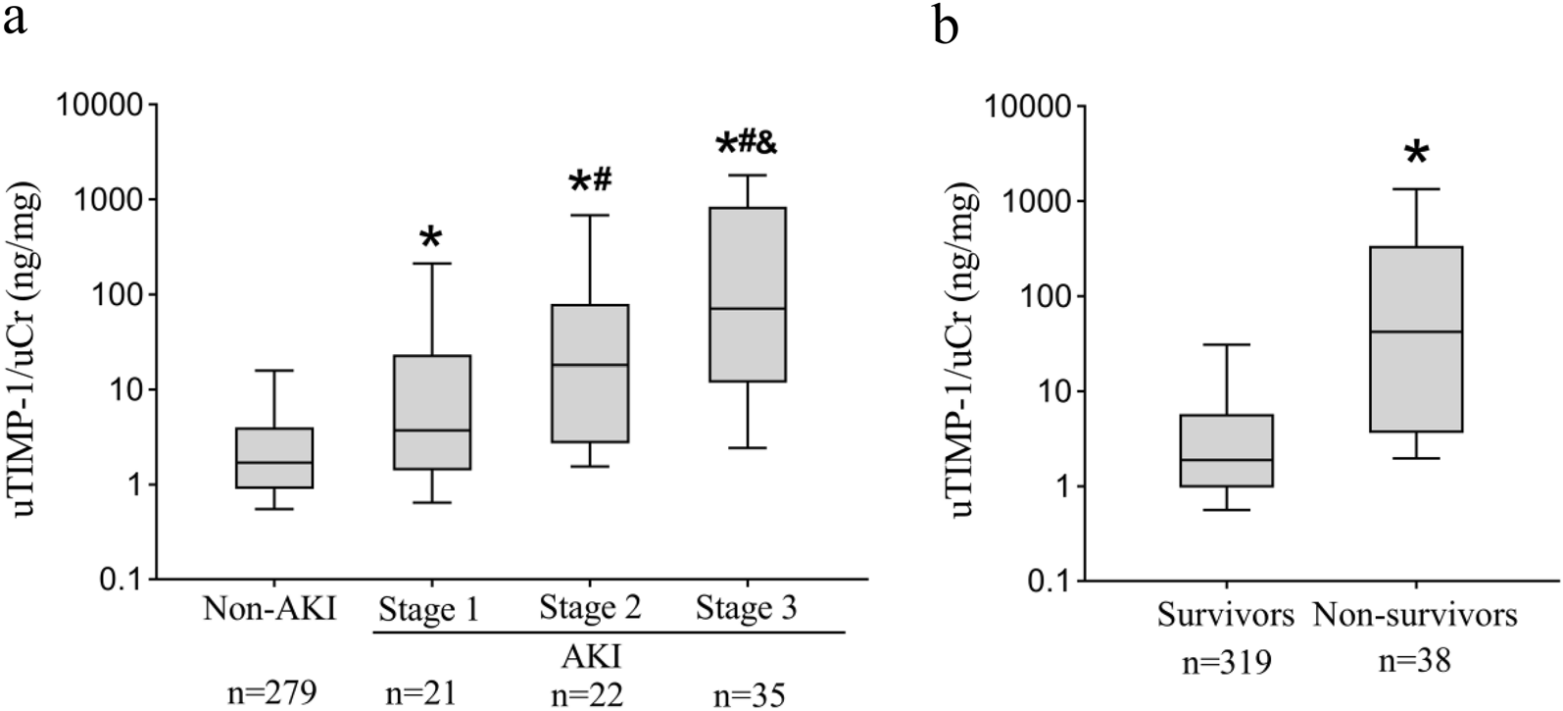
Comparisons of urinary TIMP-1 levels in the validation cohort. **a** Non-AKI and AKI status; **b** survivors and non-survivors. Lines denote median values, boxes represent 25th to 75th percentiles and whiskers indicate the range. Numbers of samples are indicated in bottom. *P < 0.05 vs. non-AKI (**a**) or survivors (**b**), #P < 0.05 vs. AKI Stage 1, &P < 0.05 vs. AKI Stage 2. *AKI* acute kidney injury, *TIMP-1* tissue inhibitor of metalloproteinases-1

Among the 357 critically ill children, 78 (21.8%) developed AKI during the first week after admission, including 21 with AKI stage 1, 22 with stage 2, and 35 with AKI stage 3. A comparison of demographic and clinical characteristics among non-AKI and AKI status in the validation cohort is depicted in Table 1. The level of uTIMP-1 was significantly higher in critically ill children with AKI than in those without AKI (1.70 [0.90–4.02] vs. 21.09 [3.14–181.02], P < 0.001). As the severity of AKI increased, the levels of uTIMP-1 were higher, as displayed in Fig. 3a.

**Table 1:** Comparison of demographic and clinical characteristics among non-AKI and AKI status in validation cohort

	Non-AKI n = 279	AKI Stage 1 n = 21	AKI Stage 2 n = 22	AKI Stage 3 n = 35	P value
Age, months	22.5 [5.5–54.0]	11.0 [2.5–44.5]	48.0 [3.9–125.0]	16.0 [7.0–55.0]^#^	0.16
Body weight, kg	12.0 [8.0–18.0]	9.0 [5.3–16.5]*	17.5 [7.6–25.8]	10.0 [7.0–16.0]^#^	0.09
Male, n	184 (65.9)	13 (61.9)	15 (68.2)	20 (57.1)	0.76
PRISM III, score	2 [0–8]	5 [3–12]*	12 [5–23]*^#^	12 [7–17]*^#^	< 0.001
MV^a^, n	61 (21.9)	6 (28.6)	12 (54.5)*	20 (57.1)*	< 0.001
Sepsis^a^, n	50 (17.9)	3 (14.3)	5 (22.7)	14 (40.0)*	0.02
MODS^a^, n	6 (2.2)	2 (9.5)	4 (18.2)*	19 (54.3)*^#&^	< 0.001
Shock/DIC^a^, n	7 (2.5)	2 (9.5)	9 (40.9)*	12 (34.3)*	< 0.001
Antibiotic^a^, n	220 (78.9)	21 (100)*	18 (81.8)	30 (85.7)	0.10
Inotrope^a^, n	14 (5.0)	2 (9.5)	10 (45.5)*^#^	12 (34.3)*	< 0.001
Furosemide^a^, n	62 (22.2)	6 (28.6)	6 (27.3)	21 (60.0)*^#^	< 0.001
Steroid^a^, n	130 (46.6)	11 (52.4)	10 (45.5)	9 (25.7)*	0.11
Hemofiltration^a^, n	9 (3.2)	2 (9.5)	2 (9.1)	11 (31.4)*	< 0.001
LOS of PICU, hours	93.0 [48.0–163.0]	125.0 [66.5–264.0]	115.5 [30.5–276.0]	132.0 [81.0–288.0]*	0.03
PICU Mortality, n	11 (3.9)	2 (9.5)	6 (27.3)*	19 (54.3)*^#^	< 0.001

*AKI* acute kidney injury, *DIC* disseminated intravascular coagulation, *LOS* length of stay, *MODS* multi-organ dysfunction syndrome, *MV* mechanical ventilation, *PICU* pediatric intensive care unit, *PRISM III* pediatric risk of mortality III

Values are median [interquartile range]. Numbers in parentheses denote percentages

^a^Administered or developed during PICU stay. *P < 0.05 vs. non-AKI, ^#^P < 0.05 vs. AKI Stage 1, ^&^P < 0.05 vs. AKI Stage 2

In the validation cohort, 38 (10.6%) died during the PICU stay. The comparison of the uTIMP-1 level between survivors and non-survivors is displayed in Fig. 3b. These characteristic comparisons between survivors and non-survivors are summarized in Additional file 1: Table S6.

### Correlation of uTIMP-1 levels with clinical variables in the validation cohort

All variables in Table 1 were analysed for association with uTIMP-1. On univariate linear regression analysis, uTIMP-1 was significantly correlated with PRISM III score, AKI stage, sepsis, MODS, shock/DIC and the use of mechanical ventilation, inotrope, furosemide, steroid and hemofiltration. To investigate factors independently associated with uTIMP-1 levels, variables with P < 0.05 under univariate analysis were entered into stepwise multivariate linear regression analysis after checking for multicollinearity. As listed in Table 2, the uTIMP-1 level was independently associated with the PRISM III score (P < 0.001), AKI stage (P < 0.001) and sepsis (P = 0.007).

**Table 2:** Univariate and multivariate linear regression analysis for clinical variables associated with initial urinary TIMP-1 level

	Univariate regression^a^			Multivariate regression^b^		
	B	SE	P value	B	SE	P value value
Age, months	0.100	0.073	0.17	N/A		
Body weight, kg	0.139	0.161	0.39	N/A		
Sex	− 0.116	0.100	0.25	N/A		
PRISM III score	0.052	0.005	< 0.001	0.031	0.006	< 0.001
AKI stage	0.489	0.041	< 0.001	0.377	0.049	< 0.001
MV	0.494	0.103	< 0.001	0.180	0.104	0.09
Sepsis	0.487	0.116	< 0.001	0.277	0.102	0.007
MODS	1.099	0.159	< 0.001	0.015	0.183	0.93
Shock/DIC	0.769	0.167	< 0.001	− 0.325	0.175	0.06
Antibiotic	0.116	0.121	0.34	N/A		
Inotrope	0.583	0.151	< 0.001	− 0.251	0.151	0.10
Furosemide	0.411	0.106	< 0.001	− 0.034	0.102	0.74
Steroid	− 0.239	0.095	0.01	− 0.110	0.081	0.17
Hemofiltration	1.021	0.182	< 0.001	0.306	0.171	0.07

*AKI* acute kidney injury, *DIC* disseminated intravascular coagulation, *MODS* multi-organ dysfunction syndrome, *MV* mechanical ventilation, *N/A* not applicable, *PRISM III* pediatric risk of mortality III. Continuous variables were log-transformed in the linear regression analyses

^a^All variables in Table 1 were analyzed in the univariate linear analysis

^b^Variables with P < 0.05 were entered into the multivariate analysis after checking the multicollinearity by variance inflation factor and tolerance values

### Association between uTIMP-1 and AKI in the validation cohort

Univariate and multivariate logistic regression analyses were performed to validate whether uTIMP-1 levels were independently associated with AKI, severe AKI, or AKI stage 3 in critically ill children in the validation cohort, as shown in Table 3. The uTIMP-1 levels remained significantly associated with AKI (AOR = 2.88, 95% CI 1.97–4.21, P < 0.001), severe AKI (AOR = 2.62, 95% CI 1.78–3.88, P < 0.001), and stage 3 AKI (AOR = 2.94, 95% CI 1.84–4.68, P < 0.001) after adjustment for body weight, sex, PRISM III score, mechanical ventilation, sepsis, MODS and shock/DIC.

**Table 3:** Association of initial urinary TIMP-1 with AKI and PICU mortality in validation cohort

	AKI	Severe AKI	AKI stage 3	PICU mortality
OR^a^ (95% CI)	3.44 (2.50–4.74)	3.39 (2.44–4.72)	3.36 (2.34–4.83)	3.07 (2.19–4.31)
P value	< 0.001	< 0.001	< 0.001	< 0.001
AOR^a,b^ (95% CI)	2.88 (1.97–4.21)	2.62 (1.78–3.88)	2.94 (1.84–4.68)	1.92 (1.11–3.30)
P value	< 0.001	< 0.001	< 0.001	0.02
AUC (95% CI)	0.80 (0.74–0.86)	0.83 (0.77–0.89)	0.84 (0.77–0.92)	0.83 (0.76–0.89)
P value	< 0.001	< 0.001	< 0.001	< 0.001
Optimal cutoff, ng/mg uCr	4.88	5.58	11.79	11.79
Sensitivity, %	71.8	80.7	77.1	71.1
Specificity, %	78.9	78.7	84.2	84.0
PPV, %	77.2	79.1	83.0	81.6
NPV, %	73.7	80.3	78.6	74.4

Severe AKI was defined as KDIGO stage 2 or 3. Urinary TIMP-1 levels were log-transformed in the logistic regression because of the variation in the concentration

*AKI* acute kidney injury, *AOR* adjusted OR, *AUC* the area under the ROC curve, *CI* confidence interval, *NPV* negative predictive value, *OR* odds ratio, *PICU* pediatric intensive care unit, *PPV* positive predictive value, *uCr* urinary creatinine

^a^Odds ratio represents the increase in risk per log increase in urinary TIMP-1 levels, ^b^Adjustment for body weight, sex, PRISM III score, mechanical ventilation, sepsis, multi-organ dysfunction syndrome, and shock/disseminated intravascular coagulation

**Fig. 4:**
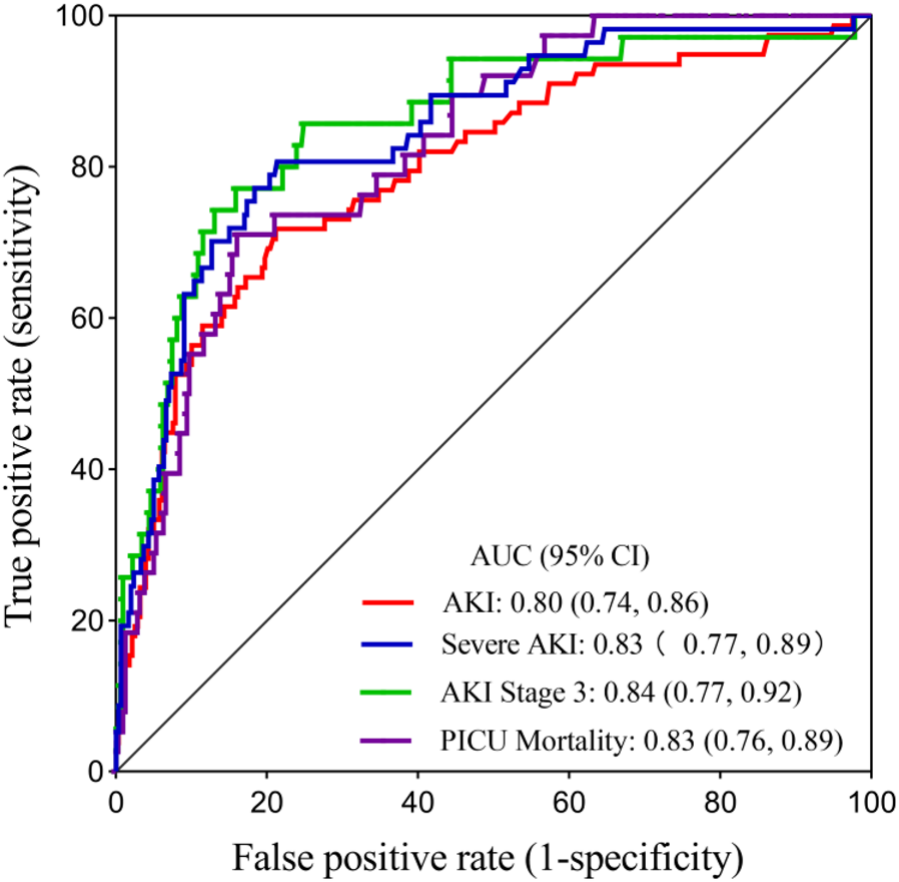
ROCs of urinary TIMP-1 to predict AKI, severe AKI, AKI stage 3 and PICU mortality. *AKI* acute kidney injury, *AUC* the area under the ROC curve, *CI* confidence interval, *PICU* pediatric intensive care unit, *ROC* receiver operating characteristic, *TIMP-1* tissue inhibitor of metalloproteinases-1

As shown in Table 3, the predictive values of uTIMP-1 for AKI, severe AKI and AKI stage 3 were 0.80 (95% CI 0.74–0.86), 0.83 (95% CI 0.77–0.89) and 0.84 (95% CI 0.77–0.92), respectively. The ROC curves for the abilities of uTIMP-1 to predict AKI, severe AKI and AKI stage 3 are displayed in Fig. 4. We also calculated the optimal cut-off values of uTIMP-1 for prediction in Table 3. Urinary TIMP-1 had a sensitivity of 71.8% and specificity of 78.9% at the optimal cut-off value of 4.88 ng/mg uCr to predict AKI.

### Association between uTIMP-1 and PICU mortality in the validation cohort

To validate whether uTIMP-1 levels were independently associated with PICU mortality in critically ill children, univariate and multivariate logistic regression analyses were performed in the validation cohort. After adjustment for body weight, sex, PRISM III score, mechanical ventilation, sepsis, MODS and shock/DIC, uTIMP-1 remained independently associated with PICU mortality (AOR = 1.92, 95% CI 1.11–3.30, P = 0.019), as listed in Table 3.

The performance of uTIMP-1 in predicting PICU mortality is also shown in Table 3 and Fig. 4. The uTIMP-1 level was predictive of mortality with an AUC of 0.83 (0.76–0.89) and had a sensitivity of 71.1% and a specificity of 84.0% at the optimal cut-off of 11.79 ng/mg uCr to predict PICU mortality in critically ill children, as shown in Table 3. The ROC curve for the ability of uTIMP-1 to predict PICU mortality is displayed in Fig. 4.

## Discussion

The diagnostic approach to AKI is currently based on sCr and urine output, which, however, do not directly reflect cell injury but rather delayed functional consequences of kidney injury. This has greatly impeded early identification and therapy. A key step for the application of novel biomarkers of AKI in clinical practice is good predictive performance with sensitivity and specificity in heterogeneous populations. In this study, the derivation and validation cohorts were performed in a mixed heterogeneous PICU. We not only detected urinary biomarkers of AKI, including NGAL, KIM-1, TIMP-2, IGFBP7, [TIMP-2]•[IGFBP7], FABP-1, TIMP-1, renin, IP-10 and TFF-3, to identify better biomarkers than others but also validated that uTIMP-1 had useful value in the early prediction of AKI and PICU mortality.

In our derivation cohort study, these urinary biomarkers had overall poorer discriminative performance in AKI stages 1 and 2, which might result from more variable urinary biomarker excretion in a PICU population of limited size. This is particularly problematic in view of the high prevalence and incidence of critically ill children with mild AKI [2]. Nevertheless, biomarkers have shown better diagnostic performance in severe AKI than in mild AKI. Severe AKI, corresponding to KDIGO stage 3, is associated with a significantly increased incidence of mortality in critically ill children [2, 22]. The PICU mortality in critically ill children with AKI stage 3 in the derivation cohort was up to 60%. Therefore, it is reasonable that we evaluated the predictive values of these urinary biomarkers for AKI stage 3 or PICU mortality in the derivation study. Our results of the derivation cohort highlight the utility and importance of initial and peak urinary TIMP-1 in critically ill children, which is similar to urinary NGAL, KIM-1 and TIMP-2 and has an increased predictive value relative to other urinary biomarkers, such as FABP-1, IGFBP7, IP-10, renin, and TTF-3, as assessed by AUCs, for the prediction of AKI stage 3 or death. In addition, a separate cohort with larger number samples was performed in turn to validate the main result from the derivation study.

Moreover, our results of the derivation cohort are consistent with previous studies conducted in children, indicating that urinary NGAL is a useful AKI biomarker for the prediction of the development of severe AKI and mortality in a heterogeneous group of patients with unknown timing of kidney injury [23, 24]. Increased urinary levels of KIM-1, FABP-1, IGFBP7, [TIMP-2]•[IGFBP7], renin and IP-10 have also been reported in children with AKI [25–31]. Urinary FABP-1 could be used for early identification of pediatric AKI in small cohorts undergoing cardiac surgery. The predictive abilities of urinary FABP-1 for AKI ranged from 0.50 to 0.87 after surgery [27, 32, 33]. The discriminative power of urinary renin for AKI stage 3 or PICU death in our present study was lower than that in our previous study [29]. The relatively small number of cases and heterogeneous PICU population in the derivation cohort study may cause these differences. Erez et al. revealed that higher urinary IP-10 concentrations were correlated with AKI in children after hematopoietic stem cell transplant [30], but they did not show the predictions. We previously demonstrated that urinary IP-10 may be a potential indicator of septic AKI and PICU mortality in critically ill children [31]. In our study, urinary TIMP-2 had better performance than urinary IGFBP7 for prediction. This difference might be explained by assuming that urinary IGFBP7 was superior to urinary TIMP-2 in surgical patients, while urinary TIMP-2 was best in sepsis-induced AKI [21]. The cell-cycle arrest biomarker urinary [TIMP-2]•[IGFBP7] is suggested to be better than any existing biomarker for predicting the development of moderate or severe AKI [21, 34]. Westhoff et al. reported that upregulated urinary [TIMP-2]•[IGFBP7] had a good performance in predicting mortality in neonatal and pediatric AKI [26]. However, the heterogeneity of the diagnostic value of urinary [TIMP-2]•[IGFBP7] for AKI has been reported and is mainly influenced by different population settings and AKI thresholds [34], which may be the most likely explanation for the lower discriminative power in our study. Our results emphasize that biomarkers of AKI must be interpreted in a specific clinical context. In addition, the measurement by using multiplex bead assays in a small sample size may be another reason for the discrepancy between our data and others. Unlike the above biomarkers, no study has evaluated TTF-3 as a urinary biomarker for predicting AKI in children. Only one study thus far has evaluated urinary TFF-3 as a biomarker of nephrotoxicity in humans [35]. Urinary TFF-3 levels were associated with death in patients with coexistent kidney disease and predicted all-cause mortality [36]. The roles of urinary TFF-3 in AKI and associated outcomes merit additional investigation.

The major finding in this study was that a higher level of TIMP-1 in urine collected during the first 24 h after PICU admission may be independently predictive of AKI and mortality developed during the PICU stay in critically ill children. To our knowledge, this is the first report of an AKI biomarker study performed in critically ill children that used a derivation-validation approach with separate patient cohorts. Urinary TIMP-1 was identified to be the better-performing marker, and we tested its performance in a second group of critically ill children. Interestingly, elevated uTIMP-1 levels showed a robust relationship with AKI and PICU mortality.

TIMP-1 is the first-discovered natural collagenase inhibitor and exhibits diverse biological functions [37]. Several studies have revealed that TIMP-1 participates in kidney injury by regulating extracellular matrix synthesis and degradation, promoting tubulointerstitial fibrosis through inhibition of proteolytic matrix metalloproteinases and exacerbating inflammation and renal scarring [38–40]. Studies on urinary TIMP-1 for AKI have mainly focused on drug-induced AKI in animal models [39–41]. To date, our report is the first clinical study to attempt to use urinary TIMP-1 as an early biomarker for AKI in critically ill children. It has been indicated that serum TIMP-1 has a higher level in septic patients with AKI and is a good diagnostic biomarker of sepsis-associated AKI [42]. In patients with sepsis after major abdominal surgery and sepsis-associated organ dysfunction, higher serum TIMP-1 levels were correlated with disease severity [43, 44], kidney injury and the use of vasopressors/inotropes [44]. Our results from the validation study further proved the independent correlations between TIMP-1 levels in urine and AKI and illness severity in critically ill children.

The positive correlation of uTIMP-1 with the PRISM III score in the study raises the question of whether uTIMP-1 levels are associated with clinical outcomes in critically ill children. Indeed, our data indicate that uTIMP-1 is an independent variable associated with PICU mortality, even after adjusting for potential confounders, including the severity of illness assessed by the PRISM III score. Compared with the discriminative ability of serum TIMP-1 for mortality in patients with sepsis [43], urinary TIMP-1 in our study had a good performance in predicting PICU mortality.

Our study has several limitations. First, the main limitation is that this was not a multicenter study. AKI occurred in the first week of PICU admission, with 60% in the first day, implying that critically ill children might be admitted later to the PICU. Nevertheless, our study is consistent with a previous study, indicating that the vast majority of children developed AKI within the first 24 h of admission to the PICU [45]. Second, since most critically ill children did not have baseline sCr prior to hospital admission, we assumed a baseline eGFR value of 120 ml/min/1.73 m^2^ as previously described [15–17]. The use of an eGFR of 120 ml/min/1.73 m^2^ as baseline might have increased the incidence of AKI in our study. Although an increasing number of equations have been developed to estimate baseline creatinine, the equations for estimating baseline creatinine differ, and the accuracy of the equations in heterogeneous PICU populations has not been clearly defined. It has been reported that compared with assuming an eGFR value of 100 ml/min/1.73 m^2^, defining an eGFR of 120 ml/min per 1.73 m^2^ as “normal” baseline renal function would be closer to true baseline renal function [17]. Third, the levels of some urinary biomarkers from the derivation cohort were slightly lower in AKI stage 2 than in non-AKI and AKI stage 1. It is possible that the relatively small number of critically ill children could have added to the large variability in the data. Fourth, we did not perform an etiological analysis for developing AKI. Since AKI is not a single disease but a complex syndrome with multiple underlying etiologies, our study was carried out in a general and mixed PICU population. It was difficult to distinguish the exact causes of AKI from the existence of complex comorbidities.

## Conclusions

Urinary TIMP-1 levels were identified and validated to be independently associated with increased risk for AKI and PICU mortality even after adjustment for confounding factors. A higher uTIMP-1 is predictive of AKI and PICU mortality in critically ill children. A large multicenter study is imperative to delineate the exact role and potential of urinary biomarkers in critically ill children.

## Supplementary Information

**Additional file 1: Table S1.** Comparison of demographic and clinical characteristics and the levels of urinary biomarkers among non-AKI and AKI status in the derivation cohort. **Table S2.** Comparison of characteristics and the levels of urinary biomarkers between survivors and non-survivors in the derivation cohort. **Table S3.** Comparison of demographic and clinical characteristics and urinary biomarkers among patients with AKI status and/or death in the derivation cohort. **Table S4.** Association of urinary biomarkers with AKI stage 3 or death developed during the PICU stay in the derivation cohort. **Table S5.** Predictive characteristics of urinary biomarkers for AKI stage 3 or death in the derivation cohort. **Table S6.** Comparison of demographic and clinical characteristics between survivors and non-survivors in the validation cohort.

**Additional file 2:** **Fig. S1**. Comparison of the initial (a) and peak (b) urinary biomarkers among patients with AKI status and/or death in the discovery cohort. Each dot represents an individual patient; the horizontal lines indicate medium with interquartile range. *P<0.05 vs. survivors with non-AKI, #P<0.05 vs. survivors with AKI stage 1 or 2. *AKI* acute kidney injury, *FABP-1* fatty acid binding protein 1, *IGFBP7* insulin-like growth factor-binding protein 7, *IP-10* interferon inducible protein-10, *KIM-1* kidney injury molecule-1, *NGAL* neutrophil gelatinase-associated lipocalin, *TFF-3* trefoil factor-3, *TIMP-1* tissue inhibitor of metalloproteinases-1, *TIMP-2* tissue inhibitor of metalloproteinases-2. Each dot represents an individual patient; the horizontal lines indicate the median with interquartile range. *P<0.05 vs. survivors with non-AKI, #P<0.05 vs. survivors with AKI Stage ½.

## Data Availability

The datasets used and/or analysed during the current study are available from the corresponding author on reasonable request.

## Abbreviations

AKI: Acute kidney injury
AOR: Adjusted odds ratio
AUC: Area under the receiver operating characteristic curve
CI: Confidence interval
Cr: Creatinine
DIC: Disseminated intravascular coagulation
FABP-1: Fattyacid-binding protein-1
ICU: Intensive care unit
IGFBP7: Insulin-like growth factor-binding protein 7
IP-10: Interferon inducible protein-10
IQR: Interquartile range
KDIGO: Kidney disease: improving global outcomes
KIM-1: Kidney injury molecule-1
LOS: Length of stay
MODS: Multiple organ dysfunction syndrome
MV: Mechanical ventilation
NGAL: Neutrophil gelatinase-associated lipocalin
NPV: Negative predictive value
OR: Odds ratio
PICU: Pediatric intensive care unit
PPV: Positive predictive value
PRISM III: Pediatric risk of mortality III
ROC: Receiver operating characteristic curve
sCr: Serum creatinine
TFF-3: Trefoil factor-3
TIMP-1: Tissue inhibitor of metalloproteinase-1
TIMP-2: Tissue inhibitor of metalloproteinase-2
uCr: Urine creatinine
uTIMP-1: Urinary TIMP-1
VIF: Variance inflation factor
