# Supplementary material for: Derivation and validation of urinary TIMP-1 for the prediction of acute kidney injury and mortality in critically ill children
Source: J Transl Med. 2022 Feb 23;20:102. doi: 10.1186/s12967-022-03302-0 (PMC8867638; doi:10.1186/s12967-022-03302-0)
Supplement: Supplementary file 1 — Additional file 1: Table S1. Comparison of demographic and clinical characteristics and the levels of urinary biomarkers among non-AKI and AKI status in the derivation cohort. Table S2. Comparison of characteristics and the levels of urinary biomarkers between survivors and non-survivors in the derivation cohort. Table S3. Comparison of demographic and clinical characteristics and urinary biomarkers among patients with AKI status and/or death in the derivation cohort. Table S4. Association of urinary biomarkers with AKI stage 3 or death developed during the PICU stay in the derivation cohort. Table S5. Predictive characteristics of urinary biomarkers for AKI stage 3 or death in the derivation cohort. Table S6. Comparison of demographic and clinical characteristics between survivors and non-survivors in the validation cohort. [file 12967_2022_3302_MOESM1_ESM.docx]

**Table S1** Comparison of demographic and clinical characteristics and the levels of urinary biomarkers among non-AKI and AKI status in derivation cohort

|  | Non-AKI | AKI Stage 1  n=16 | AKI Stage 2 | AKI Stage 3  n=5 | P value |
| --- | --- | --- | --- | --- | --- |
|  | n=94 | n=16 | n=8 | n=5 |  |
| Age, months | 14.5 [3.5-62.8] | 26.0 [7.5-105.0] | 16.0 [1.9-45.0] | 113.0 [4.5-122.5] | 0.46 |
| Body weight, kg | 10.3 [6.4-17.0] | 12.8 [8.5-23.8] | 11.0 [6.3-18.3] | 28.0 [8.0-30.5] | 0.44 |
| Sex, n | 65 (69.1) | 10 (62.5) | 6 (75.0) | 3 (60.0) | 0.84 |
| PRISM III, score | 5 [2-9] | 8 [3-14]* | 8 [6-14]^$^ | 14 [9-22]* | 0.006 |
| MV^a^, n | 37 (39.4) | 11 (68.8)* | 3 (37.5) | 3 (60.0) | 0.13 |
| MV Duration, hours | 0 [0-144.0] | 23.5 [0-167.8] | 0 [0-30.4] | 93.5 [0-793.0] | 0.24 |
| Sepsis^a^, n | 15 (16.0) | 3 (18.8) | 4 (50.0)* | 1 (20.0) | 0.12 |
| MODS^a^, n | 12 (12.8) | 5 (31.3) | 3 (37.5) | 3 (60.0)* | 0.006 |
| Shock/DIC^a^, n | 10 (10.6) | 6 (37.5)* | 4 (50.0)*^$^ | 1 (20.0) | 0.002 |
| Furosemide^a^, n | 17 (18.1) | 5 (31.3) | 1 (12.5) | 3 (60.0) | 0.10 |
| Steroid^a^, n | 51 (54.3) | 9 (56.3) | 5 (62.5) | 4 (80.0) | 0.79 |
| Antibiotics^a^, n | 80 (85.1) | 13 (81.3) | 8 (100.0) | 5 (100.0) | 0.49 |
| Inotrope^a^, n | 10 (10.6) | 4 (25.0) | 0 (0) | 1 (20.0) | 0.20 |
| Hemofiltration^a^, n | 4 (4.3) | 1 (6.3) | 0 (0) | 3 (60.0)*^&^ | 0.004 |
| LOS of PICU, hours | 103.8 [49.1-173.3] | 65.1 [35.7-301.5] | 77.3 [36.7-149.8] | 377.4 [194.6-816.6]*^#&^ | 0.03 |
| PICU Mortality, n | 8 (8.5) | 4 (25.0) | 0 (0) | 3 (60.0)* | 0.005 |
| Initial urinary biomarkers, ng/mg uCr | | | | | |
| NGAL | 34.6 [14.3-97.0] | 151.1 [15.8-1822.5]* | 98.7 [14.5-335.7] | 811.7 [599.5-1792.0]*^&^ | 0.003 |
| KIM-1 | 1.7 [0.8-4.9] | 3.9 [1.4-10.9] | 4.8 [1.4-18.7]^$^ | 7.2 [3.8-41.2]* | 0.009 |
| TIMP-2 | 8.1 [2.9-15.7] | 20.4 [6.4-72.7]* | 5.0 [4.1-8.1]^$^ | 26.0 [13.6-164.2]*^&^ | 0.004 |
| IGFBP7 | 684.0 [403.7-1222.7] | 1282.1 [513.5-2662.8] | 485.9 [168.4-760.4] | 1736.4 [1075.0-2661.3]*^&^ | 0.02 |
| [TIMP-2]•[IGFBP7]^b^ | 0.19 [0.05-0.78] | 0.45 [0.09-2.55] | 0.70 [0.04-2.57] | 0.36 [0.14-17.99] | 0.24 |
| FABP-1 | 10.9 [4.8-34.8] | 13.6 [5.3-561.7] | 8.2 [4.3-42.7] | 1073.1 [209.2-4119.7]*^&^ | 0.009 |
| TIMP-1 | 3.9 [1.9-11.6] | 22.5 [1.5-423.9] | 5.3 [3.4-68.2] | 313.3 [201.2-573.4]* | 0.001 |
| Renin | 0.16 [0.08-0.60] | 0.74 [0.12-5.60]* | 0.18 [0.10-1.78] | 7.71 [1.50-19.27]*^&^ | 0.004 |
| IP-10 | 0.09 [0.03-0.33] | 0.17 [0.03-3.06] | 0.71 [0.08-1.56] | 0.31 [0.15-5.07] | 0.12 |
| TFF-3 | 100.6 [31.4-402.3] | 159.4 [43.8-5140.8] | 27.9 [18.7-89.1] | 1057.2 [521.3-3612.1]*^&^ | 0.02 |
| Peak urinary biomarkers, ng/mg uCr | | | | | |
| NGAL | 47.1 [18.9-200.2] | 159.1 [58.6-1915.8]* | 112.7 [19.4-525.7]^$^ | 1764.4 [659.2-7557.4]*^&^ | 0.001 |
| KIM-1 | 2.8 [1.4-8.1] | 7.6 [2.3-22.9]* | 5.1 [1.4-21.0] | 18.7 [12.6-89.4]* | 0.006 |
| TIMP-2 | 12.8 [6.8-23.2] | 23.9 [8.7-106.3]* | 12.8 [4.2-25.2]^#$^ | 63.0 [45.2-231.2]*^&^ | 0.001 |
| IGFBP7 | 846.2 [496.6-1719.3] | 1702.3 [680.9-2662.8] | 889.5 [264.1-1523.9] | 4090.5 [2183.8-6396.3]*^#&^ | 0.003 |
| [TIMP-2]•[IGFBP7]^b^ | 0.58 [0.15-1.33] | 1.24 [0.55-3.38]* | 0.84 [0.07-2.73]^$^ | 1.58 [0.88-20.98]* | 0.01 |
| FABP-1 | 15.3 [7.5-55.7] | 16.1 [6.1-570.0] | 13.4 [4.3-48.4] | 1073.1 [209.2-23825.9]*^&^ | 0.02 |
| TIMP-1 | 6.3 [2.4-27.4] | 36.1 [6.2-468.7]* | 5.3 [3.4-68.2] | 605.7 [218.5-1000.4]*^#&^ | 0.001 |
| Renin | 0.27 [0.10-1.43] | 2.07 [0.49-7.00]* | 0.43 [0.12-2.18]^$^ | 7.71 [3.48-105.28]*^&^ | 0.02 |
| IP-10 | 0.11 [0.04-0.73] | 0.17 [0.04-3.21] | 0.71 [0.08-1.56] | 3.55 [1.03-6.92]*^&^ | <0.001 |
| TFF-3 | 112.3 [45.2-625.5] | 202.2 [57.0-7501.7] | 49.2 [20.8-314.4] | 3252.7 [836.7-8017.3]*^&^ | 0.02 |

Values are median [interquartile range]. Numbers in parentheses denote percentages.

^a^Administered or developed during PICU stay. ^b^[TIMP-2]•[IGFBP7] (ng/ml)^2^/1000. *P<0.05 vs. non-AKI, ^#^P<0.05 vs. AKI Stage 1, ^&^P<0.05 vs. AKI Stage 2, ^$^P<0.05 among non-AKI, AKI stage 1 and AKI stage 2.

*AKI* acute kidney injury, *DIC* disseminated intravascular coagulation, *FABP-1* fatty acid binding protein 1, *IGFBP7* insulin-like growth factor-binding protein 7, *IP-10* interferon inducible protein-10, *KIM-1* kidney injury molecule-1, *LOS* length of stay, *MODS* multi-organ dysfunction syndrome, *MV* mechanical ventilation, *NGAL* neutrophil gelatinase-associated lipocalin, *PICU* pediatric intensive care unit, *PRISM III* pediatric risk of mortality III, *TFF-3* trefoil factor-3, *TIMP-1* tissue inhibitor of metalloproteinases-1, *TIMP-2* tissue inhibitor of metalloproteinases-2.

**Table S2** Comparison of characteristics and the levels of urinary biomarkers between survivors and non-survivors in derivation cohort

|  | Survivors  n=108 | Non-survivors  n=15 | P value |
| --- | --- | --- | --- |
| Age, months | 18.5 [3.6-64.3] | 9.6 [4.5-117.0] | 0.85 |
| Body weight, kg | 11.5 [7.0-19.8] | 10.0 [7.0-25.0] | 0.69 |
| Sex, n | 33 (30.6) | 6 (40.0) | 0.56 |
| PRISM III, score | 5 [2-9] | 15 [10-29] | <0.001 |
| MV^a^, n | 39 (36.1) | 15 (100.0) | <0.001 |
| MV Duration, hours | 0 [0-86.3] | 144.0 [23.0-744.0] | <0.001 |
| AKI^b^, n | 22 (20.4) | 7 (46.7) | 0.03 |
| AKI Stage 1 | 12 (11.1) | 4 (26.7) | 0.004 |
| AKI Stage 2 | 8 (7.4) | 0 (0) |  |
| AKI Stage 3 | 2 (1.9) | 3 (20.0) |  |
| Sepsis^a^, n | 19 (17.6) | 4 (26.7) | 0.48 |
| MODS^a^, n | 13 (12.0) | 10 (66.7) | <0.001 |
| Shock/DIC^a^, n | 16 (14.8) | 5 (33.3) | 0.13 |
| Furosemide^a^, n | 20 (18.5) | 6 (40.0) | 0.09 |
| Steroid^a^, n | 59 (54.6) | 10 (66.7) | 0.42 |
| Antibiotics^a^, n | 91 (84.3) | 15 (100.0) | 0.13 |
| Inotrope^a^, n | 14 (13.0) | 1 (6.7) | 0.69 |
| Hemofiltration^a^, n | 7 (6.5) | 1 (6.7) | 1.00 |
| LOS of PICU, hours | 91.9 [46.1-170.3] | 195.0 [67.1-791.2] | 0.01 |
| Initial urinary biomarkers, ng/mg uCr | | | |
| NGAL | 34.6 [13.6-119.7] | 692.3 [107.0-1270.0] | <0.001 |
| KIM-1 | 1.8 [0.8-4.8] | 7.5 [4.0-11.2] | 0.001 |
| TIMP-2 | 7.7 [3.1-15.7] | 21.2 [14.9-29.1] | <0.001 |
| IGFBP7 | 688.9 [395.3-1304.2] | 1127.9 [618.2-2193.4] | 0.06 |
| [TIMP-2]•[IGFBP7]^c^ | 0.18 [0.05-0.77] | 1.58 [0.19-4.01] | 0.003 |
| FABP-1 | 9.9 [4.8-33.8] | 116.5 [15.5-429.9] | 0.002 |
| TIMP-1 | 3.8 [1.8-16.3] | 104.0 [18.8-313.3] | <0.001 |
| Renin | 0.17 [0.08-0.81] | 0.49 [0.16-3.14] | 0.04 |
| IP-10 | 0.09 [0.03-0.38] | 0.70 [0.18-3.23] | 0.001 |
| TFF-3 | 89.2 [29.7-388.8] | 1057.2 [101.3-6261.7] | 0.005 |
| Peak urinary biomarkers, ng/mg uCr | | | |
| NGAL | 47.3 [19.3-175.7] | 811.7 [506.7-3428.7] | <0.001 |
| KIM-1 | 2.8 [1.4-9.5] | 13.5 [8.0-18.7] | <0.001 |
| TIMP-2 | 12.1 [6.6-25.8] | 35.6 [21.2-63.0] | <0.001 |
| IGFBP7 | 884.9 [507.0-1719.6] | 2030.3 [859.1-2800.2] | 0.02 |
| [TIMP-2]•[IGFBP7]^c^ | 0.61 [0.15-1.49] | 1.58 [0.73-4.73] | 0.01 |
| FABP-1 | 13.3 [6.7-51.4] | 199.9 [37.7-13041.5] | <0.001 |
| TIMP-1 | 6.0 [2.3-28.8] | 163.2 [72.3-594.2] | <0.001 |
| Renin | 0.29 [0.11-1.82] | 2.86 [1.16-21.31] | 0.002 |
| IP-10 | 0.11 [0.04-0.69] | 3.13 [0.79-6.72] | <0.001 |
| TFF-3 | 97.8 [40.1-582.3] | 4179.1 [125.2-8926.3] | <0.001 |

Values are median [interquartile range]. Numbers in parentheses denote percentages.

^a^Administered or developed during PICU stay. ^b^Developed during the first week after PICU admission. ^c^[TIMP-2]•[IGFBP7] (ng/ml)^2^/1000.

*AKI* acute kidney injury, *DIC* disseminated intravascular coagulation, *FABP-1* fatty acid binding protein 1, *IGFBP7* insulin-like growth factor-binding protein 7, *IP-10* interferon inducible protein-10, *KIM-1* kidney injury molecule-1, *LOS* length of stay, *MODS* multi-organ dysfunction syndrome, *MV* mechanical ventilation, *NGAL* neutrophil gelatinase-associated lipocalin, *PICU* pediatric intensive care unit, *PRISM III* pediatric risk of mortality III, *TFF-3* trefoil factor-3, *TIMP-1* tissue inhibitor of metalloproteinases-1, *TIMP-2* tissue inhibitor of metalloproteinases-2.

**Table S3** Comparison of demographic, clinical characteristics and urinary biomarkers among patients with AKI status and/or death in derivation cohort

|  | Survivors | | |  | AKI stage 3  or Non-survivors  n=17 |  | P value |
| --- | --- | --- | --- | --- | --- | --- | --- |
|  | | Non-AKI  n=86 | AKI stage 1 or 2  n=20 |  |  |  |  |
| Age, months | | 15.0 [3.1-62.8] | 25.0 [4.0-59.0] |  | 12.0 [4.5-120.0] |  | 0.83 |
| Body weight, kg | | 11.0 [6.4-17.0] | 12.3 [7.3-19.8] |  | 10.0 [7.0-28.5] |  | 0.74 |
| Sex, n | | 61 (70.9) | 13 (65.0) |  | 10 (58.8) |  | 0.60 |
| PRISM III, score | | 4 [1-8] | 7 [3-12]* |  | 14 [9-29]*^#^ |  | <0.001 |
| MV^a^, n | | 29 (33.7) | 10 (50.0) |  | 15 (88.2)*^#^ |  | <0.001 |
| MV Duration, hours | | 0 [0-92.3] | 3.3 [0-80.8] |  | 93.5 [12.0-530.8]*^#^ |  | <0.001 |
| Sepsis^a^, n | | 13 (15.1) | 6 (30.0) |  | 4 (23.5) |  | 0.26 |
| MODS^a^, n | | 8 (9.3) | 5 (25.0) |  | 10 (58.8)* |  | <0.001 |
| Shock/DIC^a^, n | | 7 (8.1) | 9 (45.0)* |  | 5 (29.4)*^#^ |  | <0.001 |
| Furosemide^a^, n | | 13 (15.1) | 5 (25.0) |  | 8 (47.1)* |  | 0.01 |
| Steroid^a^, n | | 46 (53.5) | 11 (55.0) |  | 12 (70.6) |  | 0.46 |
| Antibiotics^a^, n | | 72 (83.7) | 17 (85.0) |  | 17 (100.0) |  | 0.19 |
| Inotrope^a^, n | | 10 (11.6) | 3 (15.0) |  | 2 (11.8) |  | 0.91 |
| Hemofiltration^a^, n | | 4 (4.7) | 1 (5.0) |  | 3 (17.6) |  | 0.12 |
| LOS of PICU, hours | | 91.9 [48.2-163.9] | 77.3 [35.4-226.0] |  | 195.0 [109.5-612.7]*^#^ |  | 0.02 |
| Initial urinary biomarkers, ng/mg uCr | | | | | | | |
| NGAL | | 27.2 [12.9-78.2] | 105.4 [13.6-471.5] |  | 731.4 [120.5-1792.0]*^#^ |  | <0.001 |
| KIM-1 | | 1.5 [0.8-3.9] | 2.8 [1.3-15.4]* |  | 7.5 [4.0-13.9]* |  | <0.001 |
| TIMP-2 | | 7.7 [2.6-12.1] | 7.1 [4.1-23.9] |  | 22.1 [15.5-32.4]*^#^ |  | <0.001 |
| IGFBP7 | | 675.0 [384.2-1169.7] | 713.4 [308.5-1826.7] |  | 1392.7 [687.8-2111.9]* |  | 0.07 |
| [TIMP-2]•[IGFBP7]^b^ | | 0.17 [0.04-0.69] | 0.24 [0.06-1.46] |  | 1.58 [0.27-4.88]* |  | 0.002 |
| FABP-1 | | 10.1 [4.7-30.1] | 9.3 [5.0-309.7] |  | 128.5 [26.6-1110.4]*^#^ |  | 0.001 |
| TIMP-1 | | 3.3 [1.8-8.9] | 5.6 [1.9-71.4] |  | 104.7 [22.5-342.4]*^#^ |  | <0.001 |
| Renin | | 0.15 [0.07-0.47] | 0.38 [0.10-2.70] |  | 1.16 [0.23-6.89]* |  | 0.004 |
| IP-10 | | 0.08 [0.03-0.26] | 0.21 [0.03-1.56] |  | 0.70 [0.21-2.88]* |  | <0.001 |
| TFF-3 | | 92.8 [31.4-337.2] | 67.4 [19.9-822.5] |  | 1057.1 [109.0-6126.8]*^#^ |  | 0.005 |
| Peak urinary biomarkers, ng/mg uCr | | | | | | | |
| NGAL | | 39.2 [17.8-127.9] | 151.6 [29.2-596.3]* |  | 1270.0 [557.6-4046.3]*^#^ |  | <0.001 |
| KIM-1 | | 2.7 [1.1-6.7] | 3.8 [1.7-21.0] |  | 16.1 [8.2-26.4]*^#^ |  | <0.001 |
| TIMP-2 | | 11.5 [6.4-20.8] | 19.9 [6.2-33.9] |  | 37.6 [21.6-75.2]*^#^ |  | <0.001 |
| IGFBP7 | | 817.8 [481.1-1534.9] | 1047.9 [581.1-1841.8] |  | 2030.3 [993.5-3206.4]* |  | 0.009 |
| [TIMP-2]•[IGFBP7]^b^ | | 0.54 [0.14-1.24] | 0.98 [0.40-2.74] |  | 2.51 [0.97-6.59]* |  | 0.001 |
| FABP-1 | | 12.8 [7.0-35.7] | 14.4 [5.0-309.7] |  | 339.8 [51.5-7094.6]*^#^ |  | <0.001 |
| TIMP-1 | | 5.0 [2.2-16.8] | 13.7 [3.4-71.4] |  | 217.4 [88.2-570.0]*^#^ |  | <0.001 |
| Renin | | 0.24 [0.09-0.95] | 1.54 [0.23-4.20]* |  | 3.51 [1.26-26.79]*^#^ |  | <0.001 |
| IP-10 | | 0.10 [0.04-0.42] | 0.29 [0.04-1.81] |  | 3.23 [1.03-6.02]*^#^ |  | <0.001 |
| TFF-3 | | 97.7 [40.8-441.0] | 82.4 [27.2-1534.1] |  | 3464.4 [185.1-8017.3]*^#^ |  | <0.001 |

Values are median [interquartile range]. Numbers in parentheses denote percentages.

^a^Administered or developed during PICU stay. ^b^[TIMP-2]•[IGFBP7] (ng/ml)^2^/1000. *P<0.05 vs. survivors with non-AKI, ^#^P<0.05 vs. survivors with AKI Stage 1 or 2.

*AKI* acute kidney injury, *DIC* disseminated intravascular coagulation, *FABP-1* fatty acid binding protein 1, *IGFBP7* insulin-like growth factor-binding protein 7, *IP-10* interferon inducible protein-10, *KIM-1* kidney injury molecule-1, *LOS* length of stay, *MODS* multi-organ dysfunction syndrome, *MV* mechanical ventilation, *NGAL* neutrophil gelatinase-associated lipocalin, *PICU* pediatric intensive care unit, *PRISM III* pediatric risk of mortality III, *TFF-3* trefoil factor-3, *TIMP-1* tissue inhibitor of metalloproteinases-1, *TIMP-2* tissue inhibitor of metalloproteinases-2.

**Table S4** Association of urinary biomarkers with AKI stage 3 or death developed during PICU stay in derivation cohort

|  | OR (95%CI) | P value | AOR^b^ (95%CI) | P value |
| --- | --- | --- | --- | --- |
| Initial urinary biomarkers | | | | |
| NGAL | 3.98 (2.05-7.73) | <0.001 | 2.36 (1.12-4.98) | 0.02 |
| KIM-1 | 5.20 (1.96-13.81) | 0.001 | 3.93 (1.35-11.42) | 0.01 |
| TIMP-2 | 6.53 (2.29-18.63) | <0.001 | 4.34 (1.36-13.81) | 0.01 |
| IGFBP7 | 4.11 (1.10-15.37) | 0.04 | 1.32 (0.29-6.07) | 0.72 |
| [TIMP-2]•[IGFBP7]^a^ | 3.16 (1.58-6.31) | 0.001 | 2.60 (1.15-5.88) | 0.02 |
| FABP-1 | 2.30 (1.43-3.69) | 0.001 | 1.68 (1.00-2.79) | 0.05 |
| TIMP-1 | 4.73 (2.37-9.45) | <0.001 | 3.03 (1.42-6.46) | 0.004 |
| Renin | 2.35 (1.32-4.19) | 0.004 | 1.63 (0.85-3.12) | 0.14 |
| IP-10 | 3.01 (1.58-5.73) | 0.001 | 2.27 (1.11-4.64) | 0.03 |
| TFF-3 | 2.59 (1.46-4.62) | 0.001 | 1.44 (0.71-2.92) | 0.31 |
| Peak urinary biomarkers | | | | |
| NGAL | 6.19 (2.79-13.72) | <0.001 | 4.13 (1.73-9.84) | 0.001 |
| KIM-1 | 10.41 (3.22-33.66) | <0.001 | 8.17 (2.13-31.39) | 0.002 |
| TIMP-2 | 10.19 (3.04-34.20) | <0.001 | 6.28 (1.70-23.24) | 0.006 |
| IGFBP7 | 7.52 (1.74-32.51) | 0.007 | 3.06 (0.60-15.67) | 0.18 |
| [TIMP-2]•[IGFBP7]^a^ | 3.66 (1.56-8.54) | 0.003 | 2.44 (0.92-6.43) | 0.07 |
| FABP-1 | 2.56 (1.60-4.10) | <0.001 | 2.01 (1.21-3.31) | 0.007 |
| TIMP-1 | 7.64 (3.16-18.48) | <0.001 | 5.23 (2.04-13.40) | 0.001 |
| Renin | 3.50 (1.85-6.63) | <0.001 | 2.43 (1.22-4.84) | 0.01 |
| IP-10 | 5.46 (2.47-12.05) | <0.001 | 4.28 (1.82-10.08) | 0.001 |
| TFF-3 | 3.27 (1.77-6.02) | <0.001 | 2.24 (1.08-4.66) | 0.03 |

Multivariate logistic regression analyses were performed with log-transformed data on all urinary biomarkers because of the variation in the concentration.

^a^[TIMP-2]•[IGFBP7] (ng/ml)^2^/1000. ^b^After adjustment for body weight and PRISM III score using multivariate logistic regression analyses.

*AKI* acute kidney injury, *AOR* adjusted OR, *CI* confidence interval, *FABP-1* fatty acid binding protein 1, *IGFBP7* insulin-like growth factor-binding protein 7, *IP-10* interferon inducible protein-10, *KIM-1* kidney injury molecule-1, *NGAL* neutrophil gelatinase-associated lipocalin, *PICU* pediatric intensive care unit, *TFF-3* trefoil factor-3, *TIMP-1* tissue inhibitor of metalloproteinases-1, *TIMP-2* tissue inhibitor of metalloproteinases-2.

**Table S5** Predictive characteristics of urinary biomarkers for AKI stage 3 or death in derivation cohort

|  | Initial urinary biomarkers | | |  | Peak urinary biomarkers | | |
| --- | --- | --- | --- | --- | --- | --- | --- |
|  | AUC | 95% CI | P value |  | AUC | 95% CI | P value |
| NGAL | 0.81 | 0.68-0.93 | <0.001 |  | 0.88 | 0.81-0.95 | <0.001 |
| KIM-1 | 0.78 | 0.68-0.88 | <0.001 |  | 0.84 | 0.77-0.92 | <0.001 |
| TIMP-2 | 0.82 | 0.74-0.90 | <0.001 |  | 0.83 | 0.74-0.91 | <0.001 |
| IGFBP7 | 0.67 | 0.54-0.80 | 0.03 |  | 0.72 | 0.58-0.86 | 0.004 |
| [TIMP-2]•[IGFBP7]^a^ | 0.75 | 0.63-0.87 | 0.001 |  | 0.75 | 0.61-0.88 | 0.001 |
| FABP-1 | 0.78 | 0.64-0.91 | <0.001 |  | 0.82 | 0.71-0.93 | <0.001 |
| TIMP-1 | 0.87 | 0.79-0.94 | <0.001 |  | 0.90 | 0.84-0.96 | <0.001 |
| Renin | 0.71 | 0.57-0.85 | 0.005 |  | 0.78 | 0.65-0.92 | <0.001 |
| IP-10 | 0.78 | 0.68-0.88 | <0.001 |  | 0.88 | 0.80-0.95 | <0.001 |
| TFF-3 | 0.74 | 0.61-0.88 | 0.001 |  | 0.80 | 0.70-0.90 | <0.001 |

^a^[TIMP-2]•[IGFBP7] (ng/ml)^2^/1000.

*AKI* acute kidney injury, *AUC* the area under the ROC curve, *CI* confidence interval, *FABP-1* fatty acid binding protein 1, *IGFBP7* insulin-like growth factor-binding protein 7, *IP-10* interferon inducible protein-10, *KIM-1* kidney injury molecule-1, *NGAL* neutrophil gelatinase-associated lipocalin, *TFF-3* trefoil factor-3, *TIMP-1* tissue inhibitor of metalloproteinases-1, *TIMP-2* tissue inhibitor of metalloproteinases-2.

**Table S6** Comparison of demographic and clinical characteristics between survivors and non-survivors in validation cohort

|  | Survivors  n=319 | Non-survivors  n=38 | P value |
| --- | --- | --- | --- |
| Age, months | 21.0 [4.0-58.0] | 31.5 [13.3-57.8] | 0.12 |
| Body weight, kg | 11.0 [7.0-18.0] | 13.0 [9.9-17.1] | 0.33 |
| Male, n | 207 (64.9) | 25 (65.8) | 0.91 |
| PRISM III, score | 3.0 [0.0-8.0] | 15.5 [10.5-24.5] | <0.001 |
| MV^a^, n | 66 (20.7) | 33 (86.8) | <0.001 |
| AKI^b^, n | 51 (16.0) | 27 (71.1) | <0.001 |
| AKI Stage 1 | 19 (6.0) | 2 (5.3) | <0.001 |
| AKI Stage 2 | 16 (5.0) | 6 (15.8) |  |
| AKI Stage 3 | 16 (5.0) | 19 (50.0) |  |
| Sepsis^a^, n | 55 (17.2) | 17 (44.7) | <0.001 |
| MODS^a^, n | 8 (2.5) | 23 (60.5) | <0.001 |
| Shock/DIC^a^, n | 14 (4.4) | 16 (42.1) | <0.001 |
| Antibiotic^a^, n | 255 (79.9) | 34 (89.5) | 0.16 |
| Inotrope^a^, n | 18 (5.7) | 20 (52.6) | <0.001 |
| Furosemide^a^, n | 69 (21.6) | 26 (68.4) | <0.001 |
| Steroid^a^, n | 144 (45.1) | 16 (42.1) | 0.73 |
| Hemofiltration^a^, n | 10 (3.1) | 14 (36.8) | <0.001 |
| LOS of PICU, hours | 96.0 [48.0-170.0] | 126.0 [48.0-258.0] | 0.21 |

Values are median [interquartile range]. Numbers in parentheses denote percentages.

*AKI* acute kidney injury, *DIC* disseminated intravascular coagulation, *MODS* multi-organ dysfunction syndrome, *MV* mechanical ventilation, *PICU* pediatric intensive care unit, *PRISM III* pediatric risk of mortality III.

^a^Administered or developed during PICU stay, ^b^Developed during the first week after PICU admission.
