# Supplementary figures and images for: Derivation and validation of urinary TIMP-1 for the prediction of acute kidney injury and mortality in critically ill children
Source: J Transl Med. 2022 Feb 23;20:102. doi: 10.1186/s12967-022-03302-0 (PMC8867638; doi:10.1186/s12967-022-03302-0)

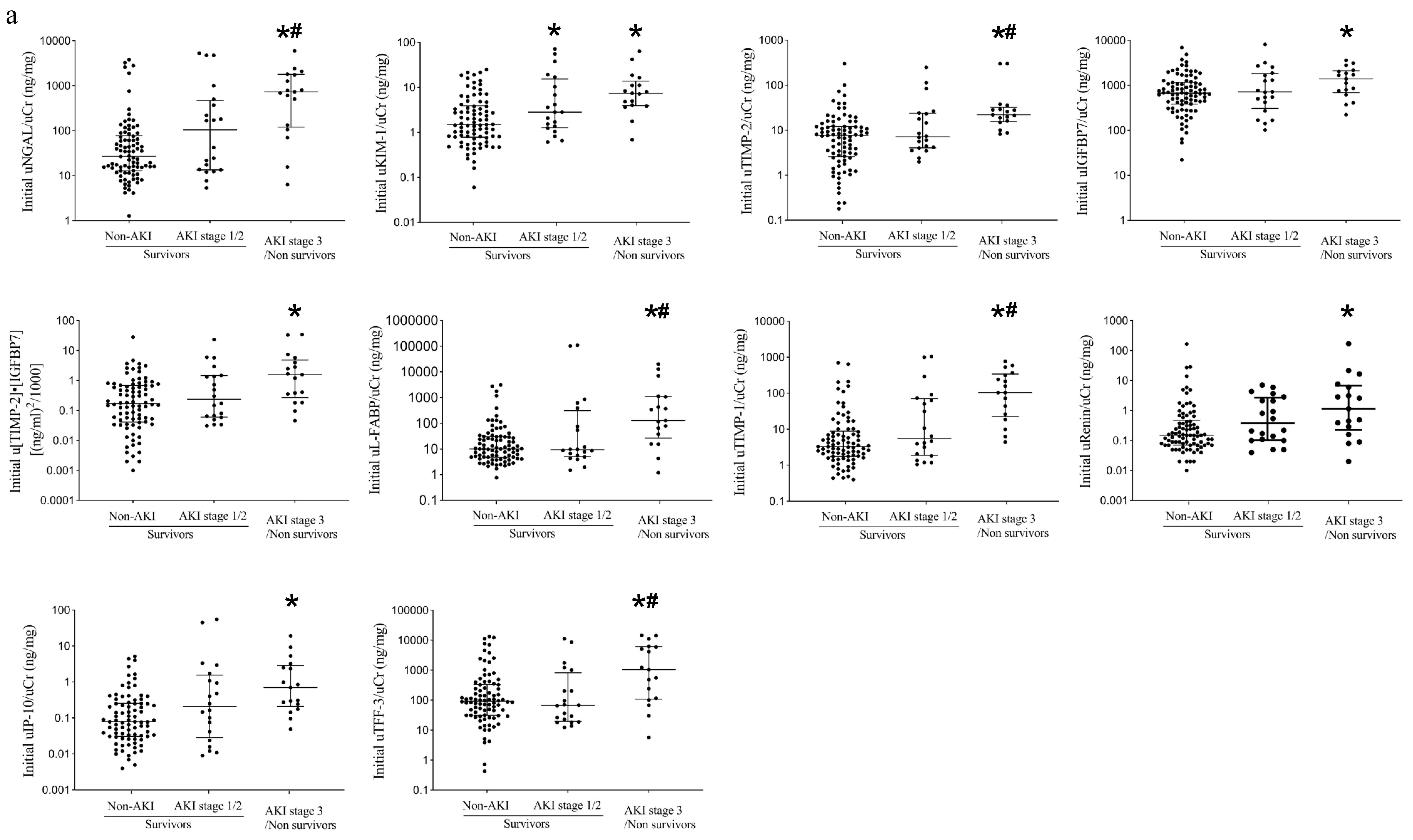

Supplement: Supplementary file 2 — Additional file 2: Fig. S1. Comparison of the initial (a) and peak (b) urinary biomarkers among patients with AKI status and/or death in the discovery cohort. Each dot represents an individual patient; the horizontal lines indicate medium with interquartile range. *P<0.05 vs. survivors with non-AKI, #P<0.05 vs. survivors with AKI stage 1 or 2. AKI acute kidney injury, FABP-1 fatty acid binding protein 1, IGFBP7 insulin-like growth factor-binding protein 7, IP-10 interferon inducible protein-10, KIM-1 kidney injury molecule-1, NGAL neutrophil gelatinase-associated lipocalin, TFF-3 trefoil factor-3, TIMP-1 tissue inhibitor of metalloproteinases-1, TIMP-2 tissue inhibitor of metalloproteinases-2. Each dot represents an individual patient; the horizontal lines indicate the median with interquartile range. *P<0.05 vs. survivors with non-AKI, #P<0.05 vs. survivors with AKI Stage ½. [file 12967_2022_3302_MOESM2_ESM.zip › figure 1a.tif]

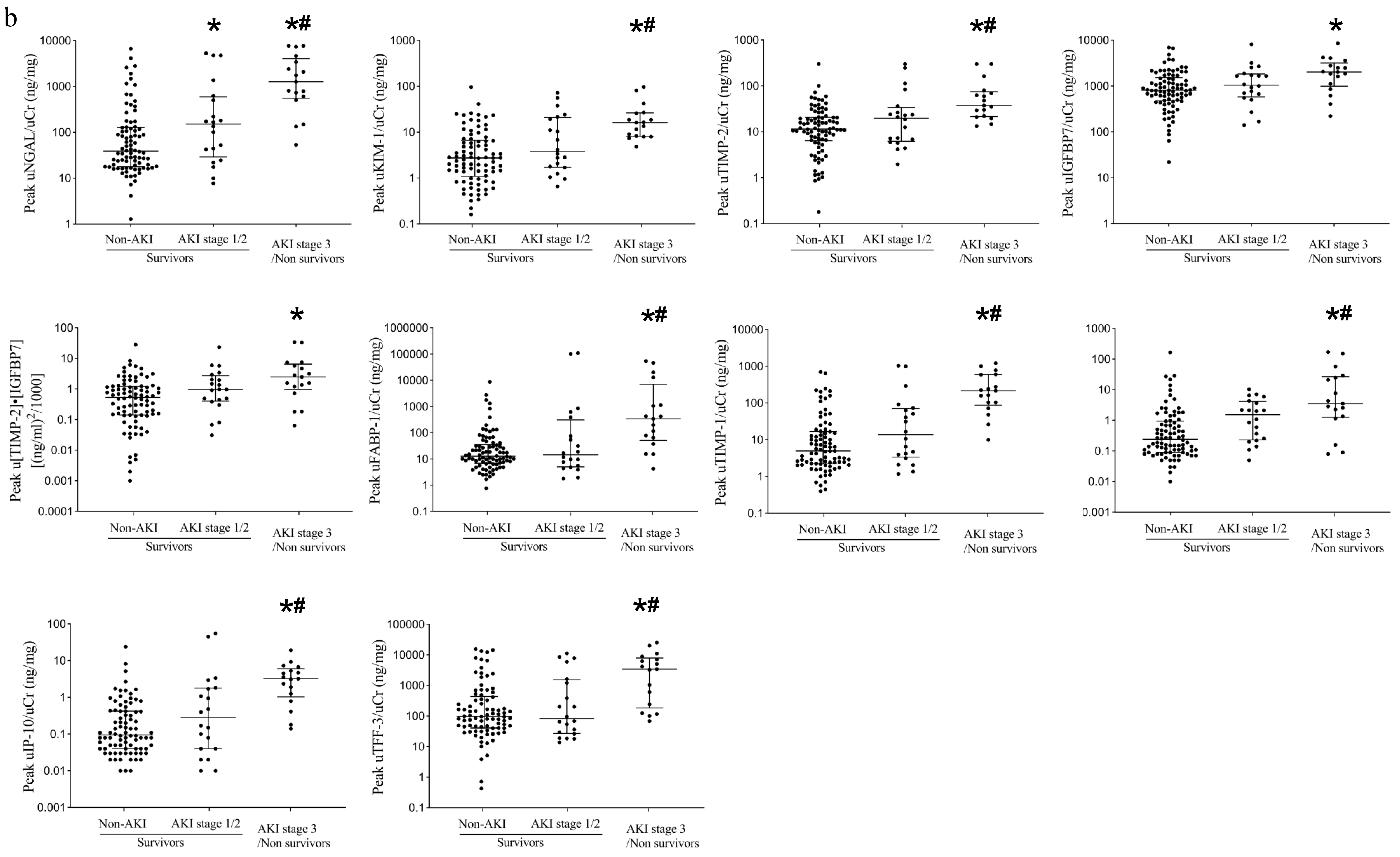

Supplement: Supplementary file 2 — Additional file 2: Fig. S1. Comparison of the initial (a) and peak (b) urinary biomarkers among patients with AKI status and/or death in the discovery cohort. Each dot represents an individual patient; the horizontal lines indicate medium with interquartile range. *P<0.05 vs. survivors with non-AKI, #P<0.05 vs. survivors with AKI stage 1 or 2. AKI acute kidney injury, FABP-1 fatty acid binding protein 1, IGFBP7 insulin-like growth factor-binding protein 7, IP-10 interferon inducible protein-10, KIM-1 kidney injury molecule-1, NGAL neutrophil gelatinase-associated lipocalin, TFF-3 trefoil factor-3, TIMP-1 tissue inhibitor of metalloproteinases-1, TIMP-2 tissue inhibitor of metalloproteinases-2. Each dot represents an individual patient; the horizontal lines indicate the median with interquartile range. *P<0.05 vs. survivors with non-AKI, #P<0.05 vs. survivors with AKI Stage ½. [file 12967_2022_3302_MOESM2_ESM.zip › figure 1b.tif]
